# Supplementary material for: Peptide Antibody Reactivity to Homologous Regions in Glutamate Decarboxylase Isoforms and Coxsackievirus B4 P2C
Source: Int J Mol Sci. 2022 Apr 17;23(8):4424. doi: 10.3390/ijms23084424 (PMC9028130; doi:10.3390/ijms23084424)
Supplement: Supplementary file 1 [file ijms-23-04424-s001.zip › ijms-1655657-supplementary.pdf]

Supplementary Materials – synthetic peptides used for peptide antibody characterization.

Immunized peptides:

GAD65: ^{250}AMMIARFKMFPEVKEKGMAALPRL^{273}

GAD67: ^{258}SIMAARYKYFPEVKTKGMAAVPKL^{281}

CVB4 P2C: ^{28}FIEWLKVKILPEVKEKHEFLSRL^{50}

Peptides used for epitope characterization of SSI-HYB 387-01:

C-terminal truncated peptides	*N-terminal truncated peptides*
SIMAARYKYFPEVKTKGMAAVPKL	SIMAARYKYFPEVKTKGMAAVPKL
SIMAARYKYFPEVKTKGMAAVPK	IMAARYKYFPEVKTKGMAAVPKL
SIMAARYKYFPEVKTKGMAAVP	MAARYKYFPEVKTKGMAAVPKL
SIMAARYKYFPEVKTKGMAAV	AARYKYFPEVKTKGMAAVPKL
SIMAARYKYFPEVKTKGMAA	ARYKYFPEVKTKGMAAVPKL
SIMAARYKYFPEVKTKGMA	RYKYFPEVKTKGMAAVPKL
SIMAARYKYFPEVKTKGM	YKYFPEVKTKGMAAVPKL
SIMAARYKYFPEVKTKG	KYFPEVKTKGMAAVPKL
SIMAARYKYFPEVKTK	YFPEVKTKGMAAVPKL
SIMAARYKYFPEVKT	FPEVKTKGMAAVPKL
SIMAARYKYFPEVK	PEVKTKGMAAVPKL
SIMAARYKYFPEV	EVKTKGMAAVPKL
SIMAARYKYFPE	VKTKGMAAVPKL
SIMAARYKYFP	KTKGMAAVPKL
SIMAARYKYF	TKGMAAVPKL
SIMAARYKY	KGMAAVPKL
SIMAARYK	GMAAVPKL
SIMAARY	MAAVPKL
SIMAAR	AAVPKL

Truncated peptides	*Alanine-substituted peptides*	*Functionality-substituted peptides*
RYKYP	AYKYFP	KYKYFP
RYKYFP	RAKYFP	RTKYFP
RYKYFPE	RYAYFP	RYRYFP
ARYKYFPE	RYKAFP	RYKTFP
ARYKYFPEV	RYKYAP	RYKYWP
	RYKYFA	

Peptides used for characterization of SSI-HYB 386-01, 386-02, 387-02, 389-01, 389-02.

GAD65 truncated peptides:	GAD67 truncated peptides:	Modified peptides:
PEVKEKGMAALPRL	EVKTGMAAVPKL	GMAA<u>A</u>PRL
KEKGMAALPRL	KTGMAAVPKL	GMAALP<u>A</u>L
KGMAALPRL	GMAAVPKL	GMAAVP<u>A</u>L
GMAALPRL	AAVPKL	AALP<u>A</u>L
AALPRL	KTGMAAVPK	
